# Physical Performance Regarding Handgrip Strength in Women with Polycystic Ovary Syndrome

**DOI:** 10.1055/s-0040-1718953

**Published:** 2020-12-21

**Authors:** Gislaine Satyko Kogure, Victor Barbosa Ribeiro, Flávia Ganoa de Oliveira Gennaro, Rui Alberto Ferriani, Cristiana Libardi Miranda-Furtado, Rosana Maria dos Reis

**Affiliations:** 1 Department of Gynecology and Obstetrics, Faculdade de Medicina de Ribeirão Preto, Universidade de São Paulo (FMRP-USP), Ribeirão Preto, SP, Brazil; 2 Instituto Federal de Educação, Ciência e Tecnologia de São Paulo, Jacareí, SP, Brazil; 3 Department of Surgery, Núcleo de Pesquisa e Desenvolvimento de Medicamentos, Universidade Federal do Ceará, Fortaleza, CE, Brazil

**Keywords:** handgrip strength, polycystic ovary syndrome, body composition, hyperandrogenism, força de preensão manual, síndrome dos ovários policísticos, composição corporal, hiperandrogenismo

## Abstract

**Objective** The present study aimed to investigate the physical performance of handgrip strength (HGS) in women with polycystic ovary syndrome (PCOS).

**Methods** A case-control study that included 70 women with PCOS and 93 age-matched healthy women aged between 18 and 47 years with body mass index (BMI) between 18 Kg/m ^2^ –39.9 Kg/m ^2^ . The serum levels of total testosterone, androstenedione, insulin, estradiol, thyroid-stimulating hormone (TSH), prolactin, sex hormone-binding globulin (SHBG), and 17-hydroxyprogesterone (17-OHP) were measured. The free androgen index (FAI) and the homeostatic model assessment of insulin resistance (HOMA-IR) were calculated. The body composition regions of interest (ROIs) were assessed by dual-energy X-ray absorptiometry (DXA), and the handgrip strength (HGS) was evaluated for both the dominant and the non-dominant hands with a manual Sammons Preston (Bolingbrook, IL, US) bulb dynamometer.

**Results** Women with PCOS had high serum levels of total testosterone ( *p <* 0.01), androstenedione ( *p* = 0.03), and insulin ( *p <* 0.01), as well as high FAI ( *p <* 0.01) and HOMA-IR ( *p* = 0.01) scores. Compared with the non-PCOS group, the PCOS group had greater total lean mass in the dominant hand ( *p* < 0.03) and greater HGS in both the dominant and the non-dominant hands ( *p <* 0.01). The HGS was correlated with lean mass ( *p <* 0.01).

**Conclusion** Women with PCOS have greater HGS. This may be associated with age and BMI, and it may be related to lean mass. In addition, the dominance effect on muscle mass may influence the physical performance regarding HGS in women with PCOS.

## Resumo

**Objetivo** O objetivo deste estudo foi avaliar a força de preensão manual (FPM) em mulheres com síndrome dos ovários policísticos (SOP).

**Métodos** Estudo de caso-controle que incluiu 70 mulheres com SOP e 93 mulheres saudáveis com idade entre 18 e 47 anos e índice de massa corporal (IMC) de 18 Kg/m ^2^ a 39.9 Kg/m ^2^ . Foram dosados os níveis séricos de testosterona total, androstenediona, insulina, estradiol, hormônio estimulador da tireoide (HET), prolactina, globulina de ligação ao hormônio sexual (GLHS), e 17-hidroxiprogesterona (17-OHP). Foram calculados o índice de androgênio livre (IAL) e a avaliação do modelo homeostático da resistência à insulina (AMH-RI). As regiões de interesse (RIs) da composição corporal foram avaliadas por absorciometria de raios-x de dupla energia (ARDE), e a força de preensão manual (FPM) das mãos dominante e não dominante foi avaliada com um dinamômetro manual Sammons Preston (Bolingbrook, IL, EUA).

**Resultados** Mulheres com SOP apresentaram níveis séricos elevados de testosterona total ( *p* < 0.01), androstenediona ( *p* = 0.03), e insulina ( *P* < 0.01), assim como valores altos no IAL ( *p* < 0.01) e no AMH-RI ( *p* = 0.01). Comparado ao grupo controle, o grupo SOP apresentou maior massa magra total na mão dominante ( *p* < 0.03) e maior FPM em ambas as mãos ( *p* < 0.01). A FPM foi correlacionada com a massa muscular magra ( *p* <0.01).

**Conclusão** Mulheres com SOP têm maior FPM, que pode estar associada à idade, ao IMC, e à massa magra. Além disso, o efeito de dominância na massa muscular pode influenciar o desempenho físico na força de preensão manual em mulheres com SOP.

## Introduction

Polycystic ovary syndrome (PCOS) is a common hormonal disorder among women of reproductive age; 1 it is characterized by elevated androgen levels, menstrual irregularities, and/or small cysts on one or both ovaries. 2 The hyperandrogenic manifestations of PCOS include dyslipidemia, insulin resistance, type-2 diabetes mellitus (DM2), obesity, cancer, infertility, and coronary heart diseases. 3 However, in almost every tissue, androgens play an essential role in many physiological processes, such as increases in protein synthesis, muscle function, and bone mineral density. Skeletal muscle is one of the main targets of androgens, which can enhance lean muscle mass and strength. 4

Handgrip strength (HGS) is a noninvasive measurement of the maximum static force that a hand can squeeze using a dynamometer. 5 It has been employed to predict overall body strength and functional performance in different groups of individuals, as well as to collect information regarding nutritional status, muscle mass, physical function, and health status. 6 7 8 A recent study 9 showed that lower relative HGS is significantly associated with a higher prevalence of metabolic syndrome in adults, indicating its long-term health implications through life.

Both HGS and skeletal muscle strength are affected by demographic data and socioeconomic variables, such as age, gender, income, and employment. Lifestyle and health behaviors, and health status or comorbidities, 10 as well as several physical factors, such as muscle mass, body mass index (BMI), 7 hand dimensions, 11 and androgens 12 are also relevant. Few studies 13 14 have investigated HGS in women with PCOS, and the results are conflicting.

We hypothesized that some phenotypic characteristics were related to HGS in women with PCOS. Therefore, the present study aimed to investigate HGS in women with and without PCOS, as well as the relationship between the physical performance of maximum voluntary strength of the hand and certain lean mass measurements.

## Methods

### Experimental Approach to the Problem

Through a secondary analysis of a case-control study, the present study sought to determine if the HGS is different ni women with PCOS and women with regular menstrual cycles. The HGS was initially analyzed for all women included in the study, and then it was paired by BMI. Additionally, through a statistical model, we sought to identify independent HGS determinants. Finally, we investigated the relationships between HGS and lean mass in the dominant and non-dominant hands. The participants were screened clinically and biochemically for enrolment into the PCOS group and the non-PCOS control group. The Review Board of the hospital of Faculdade de Medicina de Ribeirão Preto, Universidade de São Paulo (FMRP-USP), Brazil, approved the study (under process number 13475/2009). All of the ongoing and related trials have been registered at the Brazilian Clinical Trials Registry (ReBec) RBR-7p23c3.

### Subjects

Our sample consisted of 170 women in reproductive age, aged between 18 and 37 years, with normal (18 Kg/m ^2^ to 24.9 Kg/m ^2^ ), overweight (25 Kg/m ^2^ to 29.9 Kg/m ^2^ ), or obesity (> 30 Kg/m ^2^ ) BMIs according to the World Health Organization (WHO), and who had not engaged in regular and systematic physical exercise. These women were divided into 2 groups: the PCOS ( *n* = 73) and the non-PCOS ( *n* = 97) control group. They were all recruited from February 2010 to December 2013 as previously described. 13 Women with PCOS were selected at the outpatient clinics of the Sector of Human Reproduction of the Department of Gynecology and Obstetrics of FMRP-USP. The control group was recruited among women who went for routine gynecological examinations at the University Hospital and Basic Health Clinic and through public advertisements in the local newspaper and on regional television. The participants underwent transvaginal pelvic ultrasonography examinations with a Voluson 730 Expert instrument (GE Medical Systems, Zipf, Austria) to evaluate the presence of polycystic ovaries. To diagnose PCOS, peripheral blood samples were collected, and thyroid-stimulating hormone (TSH), 17-hydroxyprogesterone (17-OHP), prolactin, and testosterone concentrations were measured. Based on the results, the participants were assigned to the PCOS or non-PCOS groups. The diagnosis of PCOS was based on the presence of at least two of the following three features: chronic anovulation, hyperandrogenism (clinical or biochemical), and polycystic ovaries revealed by ultrasonography. According to the Rotterdam 2 diagnostic criteria, it is possible to identify the composition of four PCOS phenotypes: A – oligo-ovulation or anovulation + clinical and/or biochemical hyperandrogenism + polycystic ovaries; B – oligo-ovulation or anovulation + clinical and/or biochemical hyperandrogenism; C – clinical and/or biochemical hyperandrogenism + polycystic ovaries; and D – oligo-ovulation or anovulation + polycystic ovaries. Women with congenital non-classic adrenal hyperplasia, thyroid dysfunction, hyperprolactinemia, or Cushing syndrome were excluded from the study. The non-PCOS group consisted of women with regular menstrual cycles of 24 to 32 days. Individuals were excluded if they had systemic diseases, used hormonal contraceptives, smoked, or were pregnant. The women were selected regardless of race, social class, or parity. All the selected women provided a written informed consent before they were included. The baseline characteristics included age, height, weight, and BMI, which was calculated as the ratio between the weight and the height squared.

### Biochemical Measurements

The serum levels of testosterone, androstenedione, Dehydroepiandrosterone sulfate (DHEAS), and 17-OHP were measured by radioimmunoassay (Immulite1000 Immunoassay System, Siemens, Munich, Germany). Glucose levels were assessed by the glucose oxidase method. Insulin, estradiol (E2), TSH, prolactin, and sex hormone-binding globulin (SHBG) levels were assessed by chemiluminescence (IMMULITE 2000 Immunoassay System; Siemens). The free androgen index (FAI) was determined using the following equation: total testosterone (nmol/L)/SHBG (nmol/L) × 100; 15 and insulin resistance (IR) was quantified using the homeostatic model assessment of insulin resistance (HOMA-IR) ([(fasting glycemia in mg/dL x 0.05551) x fasting insulin in mU/mL]/ 22.5). 16

### Body Composition

Body composition was assessed by whole-body scan with dual-energy X-ray absorptiometry (DXA) (Hologic 4500 device, QDR Discovery Series, Hologic, Waltham, MA). The analysis was performed using the Hologic Discovery Wi software, version 13.0:5. The examination was conducted by experts at Centro de Ciências das Imagens e Física Médica (CCIFM) of FMRP-USP, following the recommendations of the International Society for Clinical Densitometry (ISCD). The regions of interest (ROIs) for the assessment were total lean mass, distribution of lean mass in the left and right arms, lean mass indices (lean mass [Kg]/height ^2^ [m ^2^ ]), and appendicular lean mass (appendicular lean mass [Kg]/height ^2^ [m ^2^ ]).

### Handgrip Strength (HGS)

The HGS test of the upper limb or upper extremity was conducted with a manual Sammons Preston (Bolingbrook, IL, US) bulb dynamometer calibrated in pounds per square inch, with measurements ranging from 10 psi to 30 psi. The HGS was measured three times per hand. One-minute intervals were allowed between the measurements, and the maximum value per hand was used for the analysis. 17 The same investigator evaluated all the patients. Hand dominance was ascertained by asking each subject which hand they used to perform well-learned skills such as writing. 18

### Statistical Analysis

All statistical analyses were performed using the PROC MIXED method of the Statistical Analysis System (SAS) software (SAS Institute Inc., Cary, NC, US) , version 9.4. The Student *t* -test was used to compare the mean variables of both groups independently, and the data are presented as mean ± standard deviation (SD). To identify the determinant variables for HGS, a multiple linear regression analysis was performed. Appropriately, group, age, BMI, the serum levels of testosterone and of androstenedione, as well as the interactions of these androgens, were considered the independent variables. The model fit was checked considering a graphical analysis of the residuals. Pearson r was used to determine the correlations between HGS and ROIs in the dominant and non-dominant hands. The level of significance was set at 5% ( *p* < 0.05) in a two-tailed test.

## Results

Of the 170 women selected for the present study, 3 women in the PCOS group and 3 in the non-PCOS group were excluded because they did not adhere to the study. Therefore, in the end, 94 women with regular menstrual cycles and 70 women with PCOS were investigated. According to the 4 PCOS phenotypes defined by the Rotterdam criteria, phenotype A was present in 34 women, phenotype B, in 5 women, phenotype C, in 19 women, and phenotype D, in 12 women. Both study groups had similar age (PCOS: 28.05 ± 5.1 years; non-PCOS: 29.5 ± 5.0 years; *p* < 0.12), height (PCOS: 160.2 ± 0.05 cm; non-PCOS: 160.1 ± 0.05 cm; *p* < 0.20), and weight (PCOS: 75.4 ± 17.6 Kg; non-PCOS: 70.4 ± 16.3 Kg; *p* < 0.05). However, the BMI was higher in the PCOS group (29.2 ± 6.5 Kg/m ^2^ ) compared with the non-PCOS group (26.9 ± 5.9 Kg/m ^2^ ) ( *p* < 0.01). Women with PCOS had increased testosterone and insulin levels, as well as higher FAI and HOMA-IR values ( *p* < 0.01, for all) compared with the control group ( Table 1 ). Concerning body composition, the total lean mass was higher in the case than in the control group ( Table 1 ).

**Table 1:** Laboratory parameters and body composition of the study sample

Variables	PCOS ( *N* = 73)	non-PCOS ( *N* = 97)	*p* -value
	[median (SD)]	[median (SD)]	
Estradiol (pg/mL)	115.3 (85.0)	120.3 (75.5)	0.71
Testosterone (ng/dL)	91.3 (36.4)	70.6 (28.7)	< 0.01
Free testosterone (nmol/L)	3.1 (1.2)	2.4 (0.9)	< 0.01
Androstenedione (ng/dL)	114.1 (34.7)	100.6 (42.1)	0.03
SHBG (nmol/L)	53.3 (38.5)	62.6 (36.5)	0.14
FAI	8.6 (5.7)	5.3 (4.0)	< 0.01
Glycemia (mg/dL)	102.0 (20.5)	97.5 (18.0)	0.15
Insulin (μIU/mL)	10.8 (13.6)	5.8 (5.1)	< 0.01
HOMA-IR	3.0 (4.3)	1.3 (1.3)	0.01
Lean mass – dominant arm (g)	2,079.5 (493.8)	1,916.8 (425.5)	0.03
Lean mass – non-dominat arm (g)	1,911.7 (557.1)	1,823.8 (392.4)	0.21
Total lean mass (g)	41,856.0 (7405.8)	36,589 (6,602.5)	0.04
Lean mass/height ^2^ (Kg/m ^2^ )	16.9 (2.8)	16.1 (2.4)	0.09
Appen. lean mass/height ^2^ (Kg/m ^2^ )	7.1 (1.3)	6.9 (1.1)	0.35

Abbreviations: Appen., appendicular; FAI, free androgen index; HOMA-IR, homeostatic model assessment-insulin resistance; PCOS, polycystic ovary syndrome; SD, standard deviation; SHBG, sex hormone-binding globulin.

**Fig. 1:**
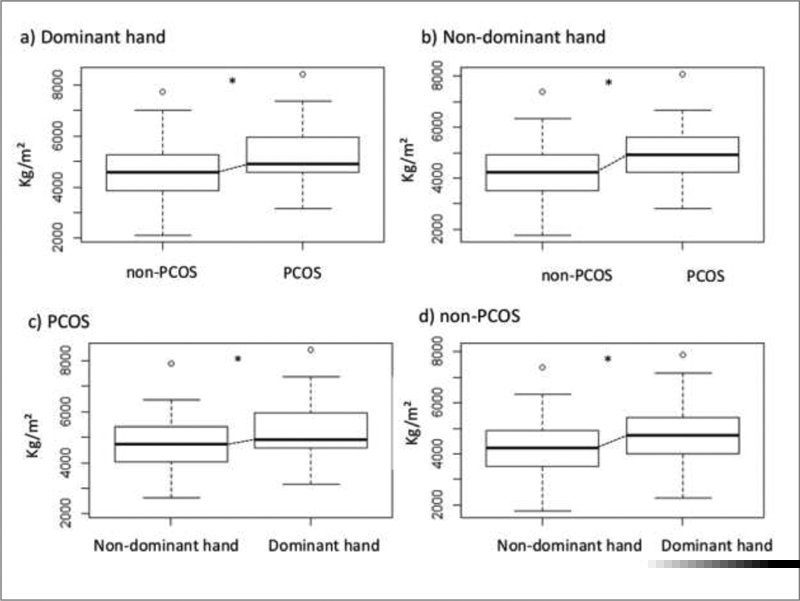
Grip strength of the dominant and non-dominant hands regarding both study groups. * *p <* 0.01.

Except for nine participants in the PCOS group and 11 participants in the non-PCOS group, the dominant hand was the right hand. None of the participants had tremor, dysmetria, or dysdiadochokinesia. In the PCOS group, the total lean mass was higher in the dominant hand ( *p* < 0.03) compared with the non-PCOS group ( Table 1 ). Fig. 1 depicts the HGS statistical analysis for both groups, in the dominant and non-dominant hands.

A total of 62 women (22 PCOS and 40 non-PCOS) had normal BMIs (18 Kg/m ^2^ to 24.9 Kg/m ^2^ ), whereas 44 women (21 PCOS and 23 non-PCOS) were overweight (25 Kg/m ^2^ to 29.9 Kg/m ^2^ ), and 58 women (27 PCOS and 31 non-PCOS) were obese (> 30 Kg/m ^2^ ). By dividing the number of women with PCOS based on the BMI, we found that overweight and obese women with PCOS had higher HGS when compared with non-PCOS women with similar BMI ( *p* = 0.01; *p <* 0.01 respectively), and that obese women with PCOS had higher lean mass/height ^2^ as compared with non-PCOS obese women ( *p* = 0.02) ( Table 2 ).

**Table 2:** Handgrip strength and body composition regarding normal weight, overweight, and obese women in the study groups

	Normal weight		Overweight		Obese	
Variables	PCOS ( *n* = 22) [median (SD)]	non-PCOS ( *n* = 40) [median (SD)]	PCOS ( *n* = 21) [median (SD)]	non-PCOS ( *n* = 23) [median (SD)]	PCOS ( *n* = 27) [median (SD)]	non-PCOS ( *n* = 31) [median (SD)]
HGS – dominant hand	4,469.4 (840.3)	4,569.8 (845.8)	5,457.0 (1,010.4)*	4,486.1 (955.6)	5,551.7 (1,004.7)*	4,817.2 (1,084.8)
HGS – non-dominant hand	4,268.5 (970.9)	4,200.3 (802.2)	4,988.3 (920.8)*	4,335.5 (1135.4)	5,236.5 (986.9)*	4,439.6 (1,059.7)
Lean mass – dominant arm	1,601.2 (214.7)	1,646.7 (244.5)	1,996.1 (286.3)	1,895.4 (305.9)	2,508.2 (386.7)	2,334.1 (386.7)
Lean mass – non-dominat arm	1,510.7 (232.5)	1,590.2 (220.3)	1,816.5 (242.1)	1,808.9 (322.6)	2,289.5 (409.2)	2,182.1 (399.1)
Total lean mass	34,649.7 (3,486.5)	34,525.7 (3,120.6)	39,760.8 (3,187.6)	39,655.0 (3,647.0)	48,930.5 (5,188.7)	4,7017.6 (5,213.9)
Lean mass/height ^2^	13.9 (1.1)	14.1 (1.1)	16.2 (1.1)	15.9 (1.2)	20.0 (1.5)*	18.8 (1.8)
Appen. lean mass /height ^2^	5.7 (0.6)	6.0 (0.6)	6.9 (0.6)	6.8 (0.6)	8.6 (0.7)	8.2 (0.8)

Abbreviations: Appen, appendicular; HGS, handgrip strength; PCOS, polycystic ovary syndrome; SD, standard deviation.

Note: * *p <* 0.05.

**Fig. 2:**
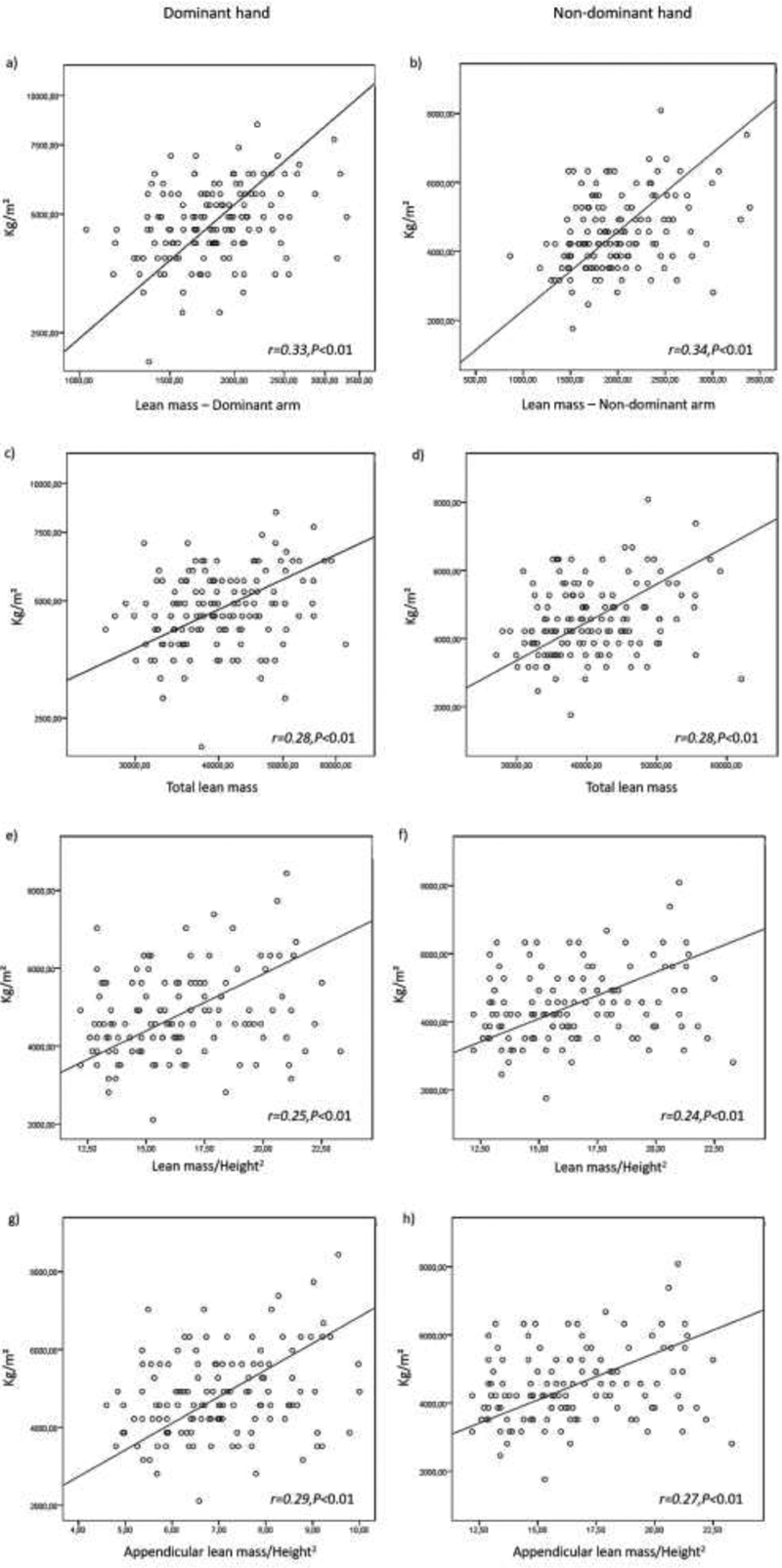
Relationship between dominant and non-dominant handgrip strength with lean mass indicators in the case group. * *p <* 0.01.

In the statistical modelling herein considered, for the whole sample, BMI and the serum level of androstenedione were predictors of HGS in both hands, but not the serum level of testosterone and the androgen interactions (testosterone and androstenedione) ( Table 3 ). According to the outcomes of the Pearson correlation tests, in the PCOS group, the HGS correlated positively with the ROIs ( *p <* 0.01) and lean mass indices ( *p <* 0.01) in both hands ( Fig. 2 ). In the non-PCOS group, there was a moderate positive correlation between HGS and lean mass in the dominant hand only (r = 0.26; *p* = 0.02).

**Table 3:** Effects of BMI, age, testosterone, androstenedione, and androgen interactions on handgrip strength

Variable	Handgrip strength									
	Dominant hand					Non-dominant hand				
	Sum of square	df	Mean square	F value	Pr > F	Sum of square	df	Mean square	F value	Pr > F
Group	7,035,308.6	1	7,035,308.6	7.81	*< 0.01*	6,110,106.1	1	6,110,106.1	7.25	*< 0.01*
Body mass index (Kg/m ^2^ )	6,350,647.2	1	6,350,647.2	7.05	*< 0.01*	9,090,583.8	1	9,090,583.8	10.79	*< 0.01*
Age (years)	2,269,183.0	1	2,269,183.0	2.52	0.11	603,112.5	1	603,112.5	0.72	0.40
Testosterone (ng/dL)	79,826.2	1	79,826.2	0.09	0.76	281,617.1	1	281,617.1	0.33	0.56
Androstenedione (ng/dL)	3,538,715.9	1	3,538,715.9	3.93	*0.04*	6,899,149.3	1	6,899,149.3	8.19	*< 0.01*
Testosterone* Androstenedione	1,308,819.3	1	1,308,819.3	1.45	0.23	1,632,776.2	1	1,632,776.2	1.94	0.16

Note: * *p <* 0.05.

Pr > F, *p* -value associated with the F statistic; df, degrees of freedom.

## Discussion

In the present study, we have investigated the HGS of the dominant and non-dominant hands, and lean mass indicators in a group of women with PCOS versus a group of healthy women. Additionally, we have explored, in the whole sample, the influence of age, BMI, and hormonal status on muscle strength, as well as the correlation between muscle strength and lean mass indicators in women with PCOS. We have observed, in agreement with a previous study, 19 that the group of women with PCOS in the present study was characterized by having higher levels of testosterone, androstenedione, and insulin, as well as higher FAI and HOMA-IR values.

In the present study, we concluded that women with PCOS have greater HGS in both hands, and that both study groups havegreater strength in the dominant hand compared with the non-dominant hand. Few studies 20 21 have analyzed muscle strength in women with PCOS with or without physical exercise. In relation to HGS, Soyupek et al. 14 showed that muscle strength did not differ between women with PCOS and age-matched healthy women. A previous study 13 by our group showed that a small sample of women with PCOS had better performance in the dominant hand only, 22 and that excess androgens in cases of PCOS, especially androstenedione, as well as the BMI, could explain the increased muscle strength. In the present study, we have observed that age, BMI and androstenedione, but not testosterone and the interaction of these androgens, were associated with HGS for the whole study sample.

Other studies suggested that age, BMI and sex account for the variances in HGS. Perna et al. 6 observed that the grip strength increased linearly in children and in a quadratic fashion among adults of both genders, and that it peaked in the age group between 30 and 39 years for both men and women, with a gradual subsequent decline. By dividing the patients into subgroups according to BMI, the difference in HGS among women with and without PCOS only occurred when obesity or overweight was present; this difference was not observed among PCOS women with normal weight. Additionally, we have observed that obese women with PCOS have significantly higher lean mass compared with obese non-PCOS controls. The high BMI might be a result of the higher percentage of skeletal muscle mass, which can largely be responsible for body heaviness, but not the percentage of fat mass, thereby resulting in greater HGS. 23 On the other hand, another study 24 verified a weak relationship between HGS and BMI, and that overall muscle function was impaired in obese individuals as compared with their non-obese counterparts, which could explain why adiposity in the obese may be associated with a lower skeletal muscle contractile capacity.

A study 7 also showed different HGS values between genders separated by BMI (low, medium, and high), and evidenced that gender was the most significant factor affecting this variable. These differences between genders may be consistent from childhood to adolescence, between young men and young women, and in different age groups. 6 The gender-related differences in strength may be attributed to the women's tendency to have lower lean body mass, 25 and the upper-body muscularity of men. 26 According to Isen et al., 27 this gender dimorphism in physical strength between men and women overcomes the differences between the genders in terms of overall body mass and height, and thus likely reflects the higher levels of androgenic hormones. 28 These hormones promote an intense physiological effect on body composition (indeed, testosterone is considered a physiological marker of the body's anabolic state and of muscle strength). 4 Recent studies have shown this association. Scharff et al. 8 observed that after one year of hormonal treatment, grip strength decreased in transwomen (treated with the anti-androgen cyproterone acetate in combination with estradiol valerate), and increased in transmen (treated with testosterone). In transmen only, the change in grip strength was associated with the change in lean body mass. Chiu et al. 12 showed, in a study with a varied sample composed of male and female participants (7,064 people), a positive correlation between serum testosterone levels and grip strength; they also showed that high testosterone levels were negatively associated with low muscle strength. Our results confirmed that lean mass is higher in women with PCOS, and that they had higher androgen levels; however, considering the whole sample, only androstenedione, but not testosterone, as well as the interaction of these androgens, was associated to HGS.

Regarding BMI, obese women with PCOS have significantly higher lean mass. Several studies have shown increased lean body mass in PCOS 29 and classic PCOS, 30 as well as higher lean mass in obese or overweight women with or without PCOS. 31 This difference is reportedly due to fat parameters and insulin, 30 but not to androgens. 29 31 Additionally, the present study showed that in PCOS women, the HGS is positively and moderately correlated with lean mass, more precisely with all the ROIs and lean mass indices. Consistent with these results, other studies showed a site-specific relationship between HGS and muscle mass. Taaffe et al. 32 found that upper-extremity muscle mass had the strongest relationship with HGS, followed by total body muscle mass. Shin et al. 33 demonstrated a positive correlation of HGS with total and appendicular lean mass when controlling for age in postmenopausal women. Several studies support the notion that HGS is positively correlated with muscle mass, especially regarding the upper extremity. Moreover, our results revealed that muscle mass influences HGS in the dominant arm in both groups. There are previous studies that found gain in muscle strength in the dominant arm. 34 The dominance effect on muscle mass has also been demonstrated to be closely related to the muscle function. Dominance naturally influences muscle mass because the dominant arm is physically more active. However, one must be bear in mind that muscle strength results from a combination of the amount of muscle mass and muscle quality. 35

Some limitations of the present study need to be considered. Although we included volunteers who did not perform regular supervised physical activity, we do not know the participants' exact exercise capacity and muscle endurance, and we have not analyzed the levels of habitual physical activity related to work and to leisure time. In this sense, the present study lacked objective measurements of certain sociodemographic characteristics and socioeconomic status, which might or might not have been important for our final model. In addition, we have not measured or evaluated hand dimensions and other specific anthropometric measurements, such as the forearm. This may have confounded the assessment of muscle performance. Moreover, differences in protocol and HGS measurements used in different studies may affect the precision and reproducibility of the HGS measurements among different study populations.

## Conclusion

Our findings indicate that, in comparison to healthy controls, women with PCOS have better physical performance with greater HGS, which may be associated with lean muscle mass. In addition, the dominance effect on muscle mass may influence the physical performance. We also suggest that age and BMI, but not hyperandrogenism, may have important implications in muscle strength. The findings extend the relationships between the physical characteristics and hormonal changes in this syndrome, and provide information about the increased functional capacity in these women, which is usually reduced in individuals with metabolic disorders. It is known that PCOS directly affects body composition, as well as endocrine, metabolic, and cardiovascular system parameters. The simple monitoring of HGS may be a promising tool to optimize, through physical and functional assessments, the multidisciplinary care and management of PCOS. Moreover, further investigations may be beneficial for a better understanding of how lean muscle mass may prevent decline in physical performance among women in reproductive age.
